# Decreased Pain Perception by Unconscious Emotional Pictures

**DOI:** 10.3389/fpsyg.2016.01636

**Published:** 2016-10-21

**Authors:** Irene Peláez, David Martínez-Iñigo, Paloma Barjola, Susana Cardoso, Francisco Mercado

**Affiliations:** ^1^Unit of Clinical Psychology, Faculty of Health Sciences, King Juan Carlos University Madrid, Spain; ^2^Laboratory of Neuropsychophysiology, Faculty of Psychology and Sciences, University of Porto Porto, Portugal

**Keywords:** emotion, pain, unconscious emotion, negative images, attentional capture

## Abstract

Pain perception arises from a complex interaction between a nociceptive stimulus and different emotional and cognitive factors, which appear to be mediated by both automatic and controlled systems. Previous evidence has shown that whereas conscious processing of unpleasant stimuli enhances pain perception, emotional influences on pain under unaware conditions are much less known. The aim of the present study was to investigate the modulation of pain perception by unconscious emotional pictures through an emotional masking paradigm. Two kinds of both somatosensory (painful and non-painful) and emotional stimulation (negative and neutral pictures) were employed. Fifty pain-free participants were asked to rate the perception of pain they were feeling in response to laser-induced somatosensory stimuli as faster as they can. Data from pain intensity and reaction times were measured. Statistical analyses revealed a significant effect for the interaction between pain and emotional stimulation, but surprisingly this relationship was opposite to expected. In particular, lower pain intensity scores and longer reaction times were found in response to negative images being strengthened this effect for painful stimulation. Present findings suggest a clear pain perception modulation by unconscious emotional contexts. Attentional capture mechanisms triggered by unaware negative stimulation could explain this phenomenon leading to a withdrawal of processing resources from pain.

## Introduction

Pain constitutes a subjective and unpleasant phenomenon that is produced by a complex relationship between a nociceptive stimulus and a number of affective and cognitive factors that modulates pain experience from the individual. At least, a part of this complexity is reflected by a non-linear relationship between the intensity of nociceptive stimulus and pain perception (Wiech et al., 2008). Thus, several psychological factors would interact with sensory components of pain modulating either painful as not painful perception (Eccleston and Crombez, 1999; Moriarty et al., 2011).

Among psychological factors modulating pain perception, the influence of emotional meaning conveyed by the stimulation used as context on the perceived pain intensity has been repeatedly demonstrated (Meagher et al., 2001; Wied and Verbaten, 2001; Rhudy et al., 2005, 2008). Specifically, whereas negative contexts increased the magnitude of pain experience (Bantick et al., 2002; Kenntner-Mabiala et al., 2008), positive ones reduced such pain perception (Lang, 1995; Rhudy et al., 2010). Experimental methods for exploring this issue have commonly consisted of the simultaneous presentation of two stimuli, a visual emotional picture or word (e.g., neutral, negative, and positive) used as a prime and a somatosensory stimulus used as a target. For example, Rhudy et al. (2005) have shown the important role of emotion on nociceptive responses, where pleasant pictures (erotica) inhibited reactions of pain but by contrast unpleasant pictures (attack) enhanced them. Similar results were found using cold-pressor test as a method for emotional pain modulation. Thereby, participants showed the highest pain tolerance scores during pleasant picture context, whereas the unpleasant context produced the lowest scores of pain tolerance (Wied and Verbaten, 2001).

Together, these findings are in line with the *motivational priming hypothesis* proposed by Lang (1995) where positive emotional events activate the appetitive part of the motivational system (which in turn activates approaching behaviors), being the defensive part of this system activated by negative events inducing defensive behaviors. The selective activation of the appetitive and aversive streams of the motivational system following negative and positive events would enhance and reduce, respectively, the response to a painful stimulus (Meagher et al., 2001; Godinho et al., 2006). Interactions between emotion and pain at a physiological level have been proposed (Weiss et al., 2003) in which emotion would play an important role in the modulation of neural indices related to pain processing (Rhudy et al., 2013). Neural systems for emotional processing and nociception seem to share physiological transmission paths; whenever a dysfunction is manifested in one of these systems, negative consequences are often seen in the other one. Comorbidities between affective (i.e., depression and anxiety), cognitive (i.e., catastrophizing) and pain processing alterations are usually observed in chronic pain patients (Lang, 1995).

Research in chronic pain patients also supports experimental evidence coming from general population showing both higher levels in behavioral (i.e., augmented pain perception; Weiss et al., 2003) and electrophysiological responses (i.e., enhanced amplitudes of laser-evoked potentials-LEPs; Dillman et al., 2000; Price, 2000) to laser-painful stimulation during the processing of negative primes. From these results, several authors have suggested that pain-related semantic primes might pre-activate neural networks favoring pain memories and pain processing (Price, 2000; Brown, 2004; Swannell et al., 2016). According to this idea, a distributed neural network for pain memories is established in each individual's biographic memory system when is exposed repeatedly to painful experiences. Such pain network strengths its connections and increases its efficacy whenever a subject is exposed to painful stimulation (Swannell et al., 2016). Therefore, two different approaches would account for the emotional influences on pain modulation: (1) the motivational priming hypotheses—to generate an emotional negative state in the subject in which pain responses would be increased—and, (2) the activation of pain memories—it is assumed that the exposure to information about pain would create memory representations that are functionally equivalent to those encoded during episodes of pain themselves—(Meerman et al., 2011). Although, emotional priming effects on the activation of pain memories are thought to be largely unconscious (Klauer and Musch, 2003; Brown, 2004), most of the research has been carried out by means affective priming paradigms involving consciously perceived stimulation.

Several theories and experimental studies have outstanding the capacity of emotional events to exercise its modulation through not conscious pathways even when stimulation is displayed for a short time period (Rhudy and Meagher, 2001; Lu et al., 2011). Masking paradigms have been commonly used to produce unawareness emotional perception, where a brief exposure stimulus (less than 50 ms) is not consciously perceived when is preceded or followed for a mask stimulus, which is presented for a longer time period (Gibbons, 2009; Balconi and Ferrari, 2012; Kongthong et al., 2013). Only few studies have investigated such unconsciously mediated pain modulation showing that emotional influences on pain modulation have been observed in both behavioral and neurophysiological investigations (Mogg and Bradley, 1998; Klauer and Musch, 2003; Brown, 2004). Meerman et al. (2011), by using a subliminal affective priming paradigm, found that pain tolerance was modulated for the type of prime (neutral, negative, positive, and pain-related) preceding nociceptive stimulation (i.e., immersion of the hand in cold water). Specifically, painful stimulation was less tolerable when pain-related subliminal words were presented. In the same line, a recent study (Brown, 2004) combining both behavioral and LEPs measures has shown that participants reported both an enhanced pain perception and higher N2 component amplitudes following the appearance of highly pain-related subliminal words compared to low pain-related ones. Although, some experimental data would be supporting thereby that affective prime stimulation could activate pain memories generating diminished pain tolerance and augmented pain perception even when emotional stimuli were not consciously processed (Brown, 2004), evidence is still far to be consistent and explaining factors should be explored.

A methodological question to take into account is related to the kind of prime stimulation. Despite a great amount of experimental evidence has clearly showed differences between pictorial and linguistic stimuli when attentional resources are allocated to them, previous studies using the subliminal affective priming procedure have relied only on affective words as prime stimulation. Specifically, verbal emotional information have been reported as less arousing that affective pictures (Bradley et al., 1998; Frühholz et al., 2011), being consequently, less capable for eliciting emotional responses than emotional pictorial stimuli (Svensson et al., 1997). Indeed, the use of images as prime stimuli would allow to a wider generalization of current findings and complement them with those obtained through experiments using stimuli other than words.

As it was mentioned, surprisingly the influence of subliminal emotional stimuli on the pain perception it has been scarcely studied up to date. Thus, present study was aimed to explore the influence of visual masking affective stimulation on the processing of a somatosensory stimulus applied by a CO_2_ laser, which it has been used in several studies showing its effectiveness for this type of studies (Iannetti et al., 2004; Mobascher et al., 2010). We expect that negative emotional pictures will produce faster and more unpleasant responses to painful stimulation compared to emotionally neutral pictures. In order to explore how other psychological factors may modulate pain perception, we additionally assessed state and trait anxiety and catastrophism (Crombez et al., 2002; Villemure and Bushnell, 2002; Kenntner-Mabiala and Pauli, 2005).

## Methods

### Participants

Fifty participants were recruited from the student population of the Faculty of Health Sciences at the Rey Juan Carlos University (36 female, and 14 men). Participants had a mean age of 21.02 years (*SD* = 14.6). They had normal or corrected to normal vision, reporting no current or prior history of chronic pain, neurological or psychiatric disorders. None of them had taken any analgesic medication or alcohol for at least 12 h before the experimental session. Participants were given a detailed explanation of the experimental procedure and they signed a written informed consent before their participation in the study. Informed consent and procedure were approved by the Rey Juan Carlos University Research Ethics Board and it followed all requirements from this committee. Once in the laboratory, and just before the beginning of the experimental session, participants completed self-reported measure of both the state and the trait forms of the State–Trait Anxiety Inventory (STAI) (Spielberger et al., 1983). This is a well-know 40-item questionnaire designed to measure state and trait anxiety. Additionally, participants also filled out the Spanish version of the Pain Catastrophizing Scale (PCS) (Sullivan et al., 1995; Olmedilla et al., 2013), in order to assess the frequency of catastrophic thoughts when they are in pain. This scale requires that the respondent rate each of the 13 items on a 5-point scale from 0 (not at all) to 4 (all the time). The total score of the PCS scale was analyzed, along with the three subscale scores assessing rumination, magnification and helplessness.

### Stimuli and procedure

**Figure 1:**
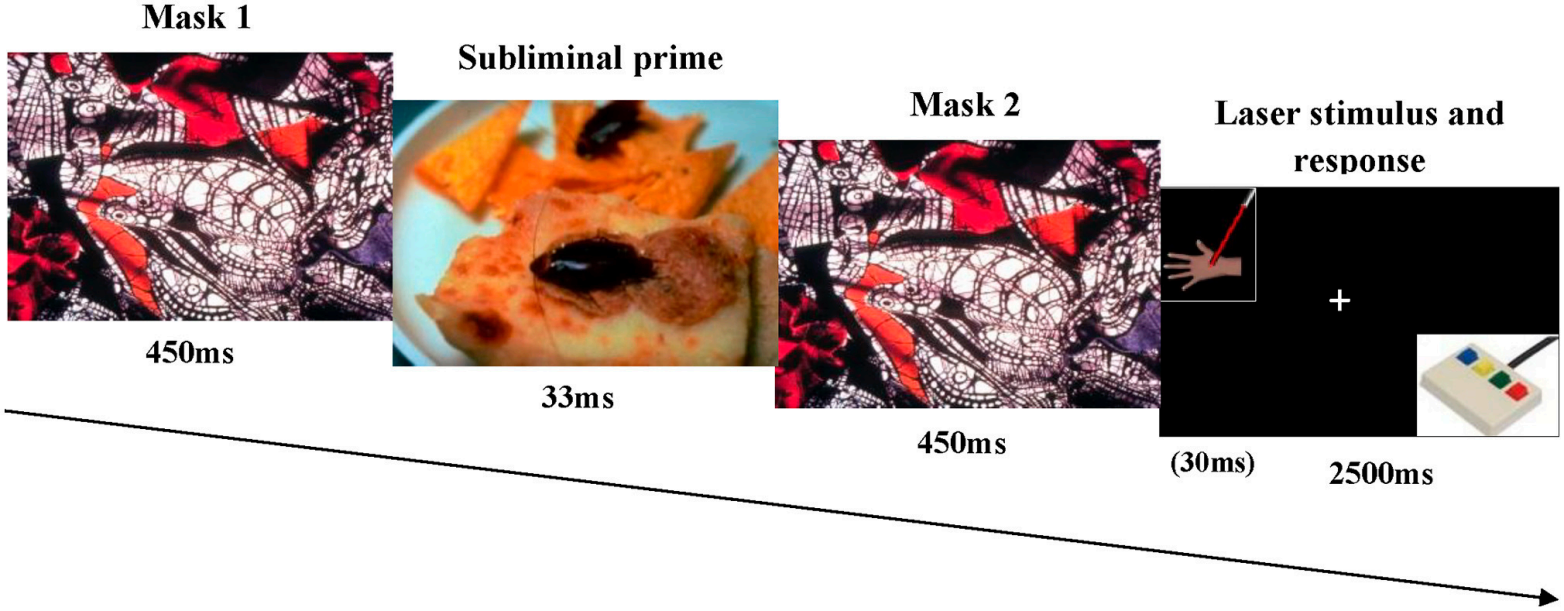
**Schematic representation of visual masking affective stimulation**. The subliminal prime (emotional image) was presented between two masks, after the last one the somatosensory stimulus was applied at the same time of the fixation point.

Stimuli presentation (i.e., masking pictures and somatosensory stimuli) and data acquisition were controlled by the Gentask module of the STIM2 package (NeuroScan Inc) which includes a dedicated visual system and a four-button response pad for data collection. During the experimental session subjects were seated in a comfortable chair in a light and sound-attenuated room facing (at a distance of 60 cm) a 19″ flat panel monitor (refresh rate 60 Hz) at eye-level which was connected to the STIM2 system. On every trial, a masked emotional picture and a somatosensory stimulus were presented in rapid succession (see Figure 1). Two types of emotional pictures were presented to the participants: arousing-negative and neutral. All images were matched in size (61, 64⋅ width × 49, 48⋅ height, visual angle degrees) and brightness. A total of 40 pictures were selected from the International Affective Picture System (IAPS) (Lang et al., 1999), 20 for each of the two emotional categories (presented twice). According to normative ratings, pictures referred to the two most important dimensions for explaining the principal variance of the emotional experience: valence (ranging from 1, negative, to 9, positive) and arousal (ranging from 1, relaxing, to 9, arousing). Picture numbers were as follows for neutral stimuli: 5510, 7000, 7002, 7004, 7006, 7009, 7025, 7041, 7050, 7059, 7080, 7090, 7100, 7150, 7175, 7224, 7235, 7242, 7491, 7950; and for negative stimuli: 1052, 1201, 1525, 1930, 2703, 2717, 2811, 3230, 6250, 6510, 6550, 6570, 6940, 7380, 9250, 9300, 9495, 9571, 9910. To ensure that emotional pictures were not consciously perceived, a forward and backward masking procedure was used. Thus, each emotional visual stimulus was presented between two mask images. Both forwards and backward masks consisted of an image that did not contain a recognizable object (see Figure 1). Despite of participants were unaware of affective stimuli presentation they were presented in semi-random order in such a way that there were never more than two consecutive trials of the same emotional category.

Each trial of the experimental task began with the presentation of a white fixation cross at the center of the screen on a black background. Participants were instructed to continuously look at that location. Then, the forward visual mask stimulus was presented for 450 ms followed by the emotional picture (33 ms). Finally, the backward mask was also presented for 450 ms. This latter stimulus was replaced by a fixation cross lasting 2500 ms. Simultaneously, to the appearance of that cross, a laser stimulus was delivered over the participant's non dominant hand. Such somatosensory stimulation was applied using a CO_2_ laser (Neurolas, Electronic Engineering; wavelength of 10.6 μm). Laser pulse was set at two intensities: infra threshold level, (non-painful stimulus: it was never perceived as painful by subjects) and supra-threshold (painful stimulus: it was always perceived as painful). Stimulation was delivered at the dorsum of the left hand being the central region avoided due to this portion has been reported as a high-sensitivity region (Iannetti et al., 2004). Stimulation area where laser pulse was delivered could be visualized through a laser beam. It was shifted about 2 cm after each trial in order to avoid nociceptor sensitization, habituation, skin damage and fatigue of that region. The hand was introduced into a box opened on the top for preventing participant could see laser beam direction and to avoid distractions. Subjects and experimenters wore protective goggles during all phases of the experimental procedure.

Before the experimental session, the two intensities for somatosensory stimulation (painful and innocuous) were determined using the method of limits, by means three stepwise series of increasing and decreasing stimulus intensities. In particular, subjects had to verbally rate the magnitude of laser stimulation by a numerical scale ranging from 0 (no sensation) to 10 (pain tolerance threshold). Beam diameter of the laser was manually ranged between 3 and 6 mm. Finally, for the experimental task, painful stimulus caused a feeling that was described from the participants as a *painful prick* (a 6 in the verbal scale), whereas innocuous stimulation was referred like *something warm* (3 in the verbal scale). For the experimental session, somatosensory stimuli were also applied in a semi-random where there were never more than two consecutive trials of the same somatosensory category. Participants were only informed that several stimulus intensities could be used during the experiment. They were asked to report the intensity of pain perceived in response to laser stimulation, as quickly as possible, pressing a button from a device with four numbers in which “1” corresponded to no pain sensation, “2” to moderate pain, “3” to intense pain, and “4” to very intense pain. A total of 160 trials (80 for each emotional category: negative and neutral), were performed in which half of somatosensory stimulus was applied above the pain threshold (painful stimulus) and the other half below pain threshold (innocuous stimulus). Therefore, 4 experimental conditions of 40 trials each were configured: negative picture followed by painful stimulus, negative picture/innocuous stimulus, neutral picture/painful stimulus and neutral picture/innocuous.

The inter-trial interval was set at 3500 ms. The task was divided into five blocks with 32 trials each and, after each block, participants were offered to an optional short break (1–2 min per break) for minimizing fatigue. Whole experimental task had a 10 min duration approximately. All participants were instructed to make a practice block to familiarize participants with experimental task. This block consisted of 20 trials containing 10 neutral images (different from those used during task) presented twice during the appearance of painful and non-painful stimuli. For all subjects, laser power and duration were set at 9 watts and 30 ms, respectively.

### Image detection test and image assessment

**Figure 2:**
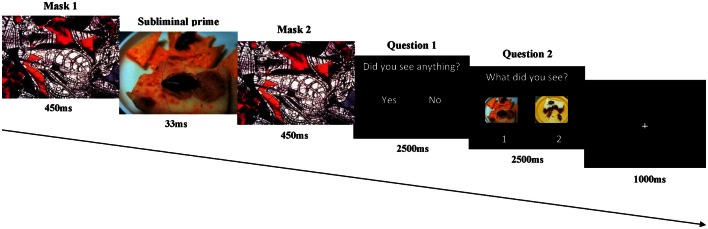
**Schematic representation of the detection image test**. The same procedure for the masking visual stimulus was used, after the last mask two questions to test the perception of the emotional images was presented.

Just after the experimental task, participants were required to perform in a forced-choice task (Gläscher and Adolphs, 2003) to check if the subliminal emotional pictures were indeed shown under the awareness threshold. Before starting the test, participants were informed about the existence of masked images. Forced-choice task was also applied by using the Gentask module of STIM2 package. Sequence of each trial was identical to the one used in the experimental task except for the two questions that were displayed to the subjects just after the visual masking stimuli: (1) the first question was “*Did you see anything?,”* in which participant's answers should be *yes*, if they had been able to distinguish some element from the masked picture, or *not*, if they could not see nothing else besides the mask; (2) and the second one was “*What did you see?.”* This question was displayed along with two different pictures, one of them was the emotional masking picture (belonging to the emotional picture set used in the experimental session) and the other one was a comparable picture in both emotional category and visual characteristics (shape and colors) to the experimental stimulation (see Figure 2). Therefore, in this test participants were instructed to say in each trial if they consciously perceived the masked picture and to decide in which location on the screen that masked picture was displayed, in the left or in the right side. Even when participant's response for the first question was negative, they also were asked to select one of the two pictures. Responses were given through the same response device used during the experimental session, but only two buttons were activated (1 = yes and left, and 2 = no and right, for the first and second question, respectively). The order of presentation of the 80 trials (20 trials for each of the two emotional categories, repeated twice, one in the left position and the other one in the right position) was pseudorandomized, so no more than three consecutive trials of the same emotional category or location were shown. The inter-trial interval was 6950 ms. Stimuli were presented in two runs of 40 stimuli with a brief resting period between them. A training block of 20 trials was provided at the beginning of the session to familiarize participants with this test. As it mentioned before, analyses on the degree of stimuli awareness were carried out. All responses given after 2500 ms and omissions were not taken into account for these analyses. Although, subjects were indeed subjectively unaware of any element included in the masked pictures (only in a 37, 1% of trials subjects said *yes* for the first question), we employed an objective threshold for unawareness defined by an identification procedure, in which if the stimulus was perceived by the subject no more than in 50% (at chance) of the cases (Merike et al., 2001), according to Signal Detection Theory (SDT) (Stanislaw and Todorov, 1999), it is unlikely that there was conscious awareness of the stimulus (*d*′ = 0).

Finally, to confirm if the pictures have the assumed valence and arousal levels for participants, they were asked to rate them on a bi-dimensional scaling test from 1-unpleasant to 5-pleasant and from 1-very relaxing to 5-very arousing respectively. Both rating scales were presented at the same time on the screen during image presentation. Participants made their ratings by selecting the option on the display with the mouse. Assessments given by the participants on these two affective dimensions of emotional stimulation are displayed in the results. For this, two one-way ANOVAs were computed for the valence and arousal of the pictures.

### Statistical analysis related to experimental effects

To test the influence of masked emotional pictures on the behavioral data, pain intensity rating (PR) and reaction times to somatosensory stimulation (RTs) were analyzed. In the case of RTs, we did outliers analyses. Responses above 2500 ms or below 200 ms, were detected in order to be omitted from the analyses. This procedure led to an average admission of 89% for negative/pain trials, 93% for negative/innocuous trials, 88% for neutral/pain trials and 91% for neutral/innocuous trials. Repeated measures ANOVAs considering RTs and PR as dependent variables and Emotion (negative and neutral) and somatosensory stimulation (painful and innocuous) as factors, were carried out. Where necessary, Greenhouse-Geisser (GG) correction was applied to adjust the degrees of freedom of the F ratios and to overcome sphericity violations. Bonferroni adjustment (alpha = 0.05) was conducted for follow-up contrasts to control for Type I error rate (reported *p*-values reflect probabilities after Bonferroni correction). A significance level of 0.05 (two-tailed) was used for all statistical analyses. Effect sizes were computed using the partial eta-square (η_*p*_^2^) method. Finally, the relationship of PR and TR with psychological factors such as Anxiety (trait and state) and Catastrophizing (rumination, magnification and helplessness), was examined by computing linear regressions. Normal distribution of the dependent variables was checked. Reaction time was normally distributed, as assessed by Shapiro-Wilks *W*-test. Pain intensity rating was asymmetrically distributed for both neutral/innocuous and negative/innocuous conditions. A −1/$\sqrt{x}$ transformation was carried out to achieve a normal distribution. As a result of this transformation, neutral/innocuous condition achieve a symmetrically distribution (*z* = 1.84) according with the sample size (*z* = 3.2). Pain intensity ratings for the negative/innocuous condition obtained a value closed to the symmetry (*z* = 3.64). Considering that repeated-measured ANOVAs are considerably robust to normality deviation we decided to compute parametric analysis^1^. All statistical analyses were carried out using IBM SPSS Statistics (version 22).

## Results

### Control analyses

Assessments given by the participants on valence and arousal for the emotional images were analyzed through repeated-measures ANOVAs for the two mentioned variables, using pictures (two levels: negative and neutral) as a factor. ANOVAs yielded significant differences in both valence [*F*_(1, 49)_ = 1160.648, *p* < 0.001, η_*p*_^2^ = 0.959] and arousal [*F*_(1, 49)_ = 756.134, *p* < 0.001, η_*p*_^2^ = 0.939], indicating that negative pictures were assessed as more negative (in the valence scale) and more arousing (in the arousal scale) than neutral pictures. Such results indicated that these stimuli were suitable for further analyses. Data about mean values for valence and arousal of the two emotional categories are displayed in Table 1. For the image detection test, the SDT parameter *d*′ was calculated for each participant before calculating the hit and false alarms rates. Mean of whole sample was *d*′ = −1.12. As it mentioned, this value indicated non-awareness discrimination for masked pictures.

**Table 1:** **Mean valence and arousal ratings with standard deviation for all slide types**.

	**Neutral**	**Negative**
Valence	3.12 (0.22)	1.41 (0.29)
Arousal	2.85 (0.27)	4.50 (0.36)

*The scores range from 1 (low pleasure, low arousal), to 5 (high pleasure, high arousal)*.

To check if there were either sensitizations or habituation effects, we carried out repeated-measures ANOVAs for each somatosensory condition (painful and innocuous) using the ratings of pain perception for each block (5 in total) as factor. We did not found differences for any block [painful: *F*_(4, 46)_ = 1.47, *p* = 0.216, η_*p*_^2^ = 0.216; innocuous: *F*_(4, 46)_ = 1.48, *p* = 0.214, η_*p*_^2^ = 0.142].

### Experimental effects

**Figure 3:**
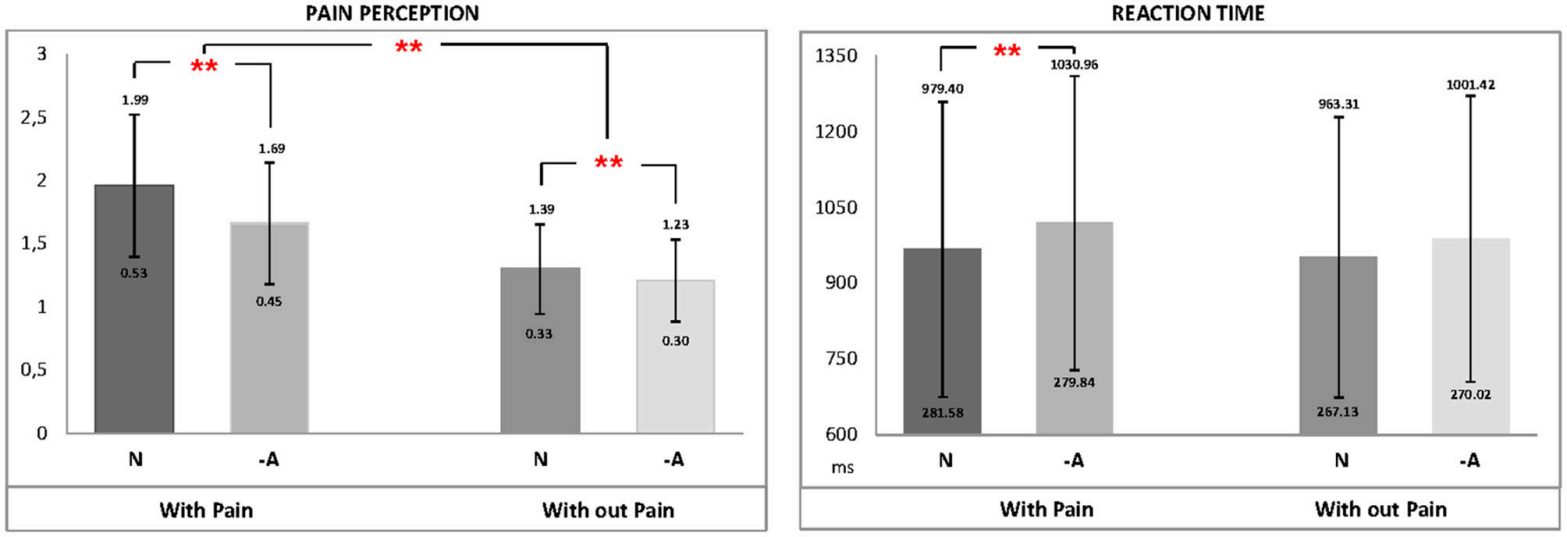
**Main pain ratings and reaction time with standard errors separate for all picture contents and painful and non-painful stimuli**. The scores ranged from 1 (no pain) to 4 (very intense pain). ^**^p < 0.01.

Mean values for PR and RTs related to both emotional picture and type of somatosensory stimulation can be seen in the Figure 3. We carried out two-way repeated-measures ANOVAs 2 × 2 on the two mentioned variables (PR and RTs). Significant main effects on PR were found for both factors emotional picture [*F*_(1, 49)_ = 54,068, *p* < 0.001, η_*p*_^2^ = 0.525] and type of somatosensory stimulation [*F*_(1, 49)_ = 135,279, *p* < 0.001, η_*p*_^2^ = 0.734]. Specifically, trials including masked negative pictures (*M* = 1.46, *SD* = 0.06) produced lower scores for PR compared to neutral ones (*M* = 1.65, *SD* = 0.05). As it expected, painful stimuli (*M* = 1.84, *SD* = 0.07) elicited higher PR than non-painful stimulation (*M* = 1.27, *SD* = 0.04). We also found a clear and significant effect [*F*_(1, 49)_ = 28,549, *p* < 0.001, η_*p*_^2^ = 0.368] for the interaction between emotional picture and somatosensory stimulation. *Post-hoc* comparisons (Bonferroni; *p* < 0.05) showed that the increase in pain perception for painful compared to non-painful stimulation was smaller when participants were exposed to masked negative pictures compared to neutral images, as it can be seen in Figure 3.

With respect to RTs, statistical analysis reached also a significant main effect for emotional picture [*F*_(1, 49)_ = 8.40, *p* < 0.01, η_*p*_^2^ = 0.146]. Specifically, trials linked to negative pictures (*M* = 1016.19, *SD* = 38.58) showed longer RTs than neutral ones (*M* = 971.36, *SD* = 37.28). However, a main effect linked to the type of somatosensory stimulation was not found [*F*_(1, 49)_ = 2.985, *p* = 0.09, η_*p*_^2^ = 0.057]. Finally, statistical analyses did not revealed either interaction effects between the two single factors for RTs [*F*_(1, 49)_ = 0.426, *p* = 0.52, η_*p*_^2^ = 0.009, see Figure 3].

In the same way, regression analyses were carried out in whole sample of participants, however, no significant predictors for behavioral indices (PR and RTs) were found among trait anxiety (β = −0.19, *p* = 0.19, *B* = −0.003, *SE* = 0.003; β = −0.21, *p* = 0.15, *B* = −2.30, *SE* = 1.56), state anxiety (β = −0.08 *p* = 0.59, *B* = 0.906, *SE* = 1.64; β = −0.013, *p* = 0.93, *B* = −0.149, *SE* = 1.63), the total score in the PCS (β = −0.04, *p* = 0.78, *B* = −0.001, *SE* = 0.003; β = 0.053, *p* = 0.71, *B* = 0.637, *SE* = 1.723) and its three subscales: rumination (β = −0.11, *p* = 0.43, *B* = −0.002, *SE* = 0.002; β = −0.05, *p* = 0.71, *B* = −0.551, *SE* = 1.48), magnification (β = 0.04, *p* = 76, *B* = 0.001, *SE* = 0.003; β = 0.17, *p* = 0.23, *B* = 1.96, *SE* = 1.63) and helplessness (β = 0.02, *p* = 0.9, *B* = 0.000, *SE* = 0.003; β = 0.06, *p* = 0.66, *B* = 0.816, *SE* = 1.86).

## Discussion

Present study was aimed to investigate the influence of subliminal emotional pictures (negative and neutral) on the pain perception through a masking affective paradigm. Affective modulation to pain perception was measured on two behavioral responses (pain intensity ratings and reaction times) linked to the processing of two types of somatosensory stimuli (painful and innocuous) delivered by a CO_2_ laser. Main results revealed that pain perception was modulated by the affective content conveyed by pictures showing that negative images generated both a lower pain perception and slower reaction times compared to the neutral ones, even when they were not consciously perceived. This prominent effect was strengthened in response to painful somatosensory stimuli. No modulation effects were found for anxiety or catastrophizing variables. As expected, our data reflect a clear emotional priming effect on pain perception, but they are in contradiction with previous experimental evidence where conscious emotional processing of negative pictures has been related to enhanced pain perception and diminished pain tolerance (Meagher et al., 2001; Rhudy et al., 2005; Godinho et al., 2006; Kenntner-Mabiala et al., 2008; Kongthong et al., 2013). Nevertheless, it should be noted that emotional modulation on pain perception is not an unequivocal finding and that this issue is still a controversial when subliminal affective priming procedures are applied (Meerman, 2012; Meerman et al., 2013). Additionally, behavioral measures related to reaction times for painful stimulation not always have been analyzed in previous studies. We will carefully discuss functional interpretation of the present data in relation to different aspects concerning the nature of both emotional stimulation and the experimental task, as factors that could contribute for explaining our results.

Experimental evidence derived from the scarce investigations about subliminal influences of emotion on pain perception is mixed and contradictory. Some studies have found that the exposure to pain-related primes associated with higher pain perception in response to painful laser stimuli, although this effect could be consciously mediated, at least partially (Balconi and Ferrari, 2012). However, this effect is not a straightforward finding. Meerman et al. (2011, 2013) and Meerman (2012) did not find consistent evidence of subliminal affective modulation on pain in a series of studies using words as emotional primes. Thus, whereas lower pain tolerance was obtained in its first experiment as a consequence of the subliminal exposure to health complaint primes (Meerman et al., 2011), in subsequent studies they did not find evidence of affective modulation (Meerman, 2012). Thus, even they have found an opposite relationship between affective subliminal words and pain tolerance, where a subsample of participants reported higher pain tolerance instead (Meerman et al., 2013). These results suggest that non-conscious emotional modulation on pain perception could depend on different factors such as individual characteristics (i.e., dispositional self-focused attention; Meerman et al., 2013) or it may be related to the type of stimulation used as emotional context or primes (i.e., words, images, etc.; Hinojosa et al., 2015). In this sense, it has been suggested that words are not capable enough to activate pain memories, because they are not representing the threatening content conveyed by the stimulation as effectively as emotional pictures are depicting for example, blood and serious wounds (Gläscher and Adolphs, 2003). Indeed, pictorial non-word stimuli has been reported as more arousing and intrusive than words (Stanislaw and Todorov, 1999; Bernat et al., 2001; Gläscher and Adolphs, 2003) being consequently more capable for capturing attention (Meerman, 2012; Meerman et al., 2013), and eliciting attentional biases to them and modulating the processing of further stimulation. Along with the fact that pictures are better able to capture attention than words, certain features of these stimuli such as its emotional meaning might also be a key component for attracting additional attentional processing resources and interfering with a given ongoing task (Carretié, 2014). This idea is in line with other investigations showing that focusing on the content of distractive and surrounding emotional events (Rhudy et al., 2005) may lead to a reduced pain perception (Kenntner-Mabiala and Pauli, 2005; Wiech et al., 2008). In the same way, the amount of attentional resources required to perform in a daily activity may modulate pain perception in such a way that distraction on pain will be more pronounced when attentional resources devoted to the task are higher (Villemure and Bushnell, 2002). Thus, Reicherts et al. (2013) studied the modulator effect of emotional faces on pain showing that participants reported decreased pain ratings under a painful thermal condition while they were watching videos displaying any emotional facial expressions compared to a control condition. This reduction of pain perception seem to be induced by both complexity and affective meaning of visual stimuli which would withdraw attention or distract from pain leading to a diminished pain report (Villemure and Bushnell, 2002; Wiech et al., 2008).

So far, it has risen as how attention can be a pain-modulating component through a conscious processing. However, it is also important to points out that stimuli processed without conscious awareness are also able to capture attention. Several studies focused on the analysis of skin conductance or cardiovascular measures in response to unaware stimuli, have associated these psychophysiological indices with attentional capture and automatic responses (Carretié et al., 2011). As it has been suggested, subliminal processing depends on some pre-attentional components that could serve as adaptive mechanisms generating an immediate response to a relevant or potentially threatening stimulus, which could occur even before its conscious evaluation (Reicherts et al., 2013). For instance, it has been indicated that subliminally-presented spatial cues were effective for capturing attention to its location and they modulated further responses to target stimuli (Eimer and Kiss, 2007; Chou and Yeh, 2011). Capture of attention have been also showed by masked affective stimuli, (Monk et al., 2008) using fMRI while participants had to perform in an attention-orienting task with masked emotional faces (angry, happy, and neutral). The results indicated that when angry faces were subliminally presented participants showed an initial attentional bias toward the spatial location of threat. Keeping this experimental evidence in mind, both the longer RT and the lower PR registered for painful stimulation when negative pictures were displayed compared to neutral ones (103,096 vs. 97,940 ms and 1.69 vs. 1.99 pain scores, respectively) suggests that emotional negative valence of stimuli recruited a great amount of processing resources (capturing attention toward it) reducing pain perception and interfering pain processing, even when it was not consciously perceived.

Therefore, we propose that one possible explanation for the experimental effects obtained in the present study would be related to a process of attentional capture. Attentional capture is an adaptive mechanism that can detect and process salient and biologically important stimuli, even if they are out of our attentional focus (bottom-up process; Öhman, 1999; Öhman et al., 2001). Thereby, endogenous or voluntary attention can be inadvertently redirected to another stimulus capturing attention and distracting from the original task. Different kind of distractors may cause disruption in the ongoing task, which is reflected in a poor processing of targets: longer RTs and/or higher error rates (Carretié, 2014). Furthermore, according to our results, it has been observed that not all stimuli have the same capacity for distracting attention (Meerman, 2012) being its effect mediated by the novelty of the stimulus (unexpected capture more attention), complexity and the stimulus emotional meaning. Research on this topic usually have shown that emotional stimuli, and more efficiently in the case of the negative pictures (more than emotional words), capture a higher amount of attentional resources than neutral events distracting the subjects from the main task and inducing a decrease in pain perception (Öhman, 1999; Stanislaw and Todorov, 1999; Öhman et al., 2001; Villemure and Bushnell, 2002; Wiech et al., 2008; Reicherts et al., 2013).

Present study provides relevant data about the power of subliminal emotional stimuli for capturing attention and reducing pain perception. Due to the scarce experimental evidence on this topic, further investigation should be done to identify in greater depth underlying mechanisms by which the unconscious processing of emotional stimuli can modulate pain through the interaction of both emotional and attentional processes, as it was already described in the case of the consciously perceived stimulation. However, some limitations of this study need to be addressed. We only used one emotional stimulus category (negative), being interesting to explore the influence of other categories (e.g., with emotional positive meaning) on pain perception. Additionally, due to the experimental paradigm required from subjects as rapid responses as they can to rate their somatosensory perceptions, device used for this task only included four response options. This setting could limit the accuracy of ratings for painful stimulation being recommendable to explore other settings to wide the number of response options. Other aspects related to visual stimulation, as such complexity of subliminal emotional pictures or task difficulty should be explored as potential factors competing for attentional resources along with pain processing. Furthermore, the application of brain functioning measures such as event-related potentials or functional magnetic resonance would allow us to investigate both neural networks and mechanisms involving in these unconsciously mediated attentional processes. Finally, more research on unconscious processing of emotional stimuli in people with chronic pain diseases should done since it could give clues to better understand pain processing disturbances in these clinical conditions, as it is the case in fibromyalgia (Mercado et al., 2013), facilitating the design of more effective treatments to manage pain symptoms.

To sum up, our research provides new evidence on the unconscious processing of emotional stimuli and its influence on the pain perception. Obtained data have shown that the subliminal exposure to threatening pictures induced both lower pain perception and slower reaction times being more clearly appreciated in response to painful stimulation. Attentional capture processes could account for such affective modulation on pain in healthy people through a distractive mechanism where threatening pictures withdraw attentional resources from pain even under subliminal conditions. The lack of studies on this issue along with inconsistent previous findings make necessary more investigation to confirm present results and to complement them with data coming from brain activity methodologies since emotional unconscious influences might depend on different neural paths than those involved under conscious situations. These findings might help us to disentangle the relationship between emotion and pain perception giving some clues to improve understanding about the unconscious emotional influences on pain processing in chronic pain patients.

## Author contributions

IP: Substantial contributions to the conception, design of the work, the acquisition, analysis, and interpretation of data for the work. Write the work. DM: Substantial contributions to the analysis, and interpretation of data for the work. Revise the work. PB: Substantial contributions to the acquisition and revise the work. SC: Substantial contributions to the acquisition. FM: Substantial contributions to the conception, design of the work, analysis, and interpretation of data for the work. Revise the work. Final approval of the version to be published.

### Conflict of interest statement

The authors declare that the research was conducted in the absence of any commercial or financial relationships that could be construed as a potential conflict of interest.

## Footnotes

^1^Non-parametric tests (Friedman ANOVAs) were carried out to confirm the results obtained from parametric tests. These analyses showed same experimental effects those parametric analyses (repeated-measures ANOVAs).
