# Corrigendum: Decreased Pain Perception by Unconscious Emotional Pictures

**DOI:** 10.3389/fpsyg.2018.01310

**Published:** 2018-08-21

**Authors:** Irene Peláez, David Martínez-Iñigo, Paloma Barjola, Susana Cardoso, Francisco Mercado

**Affiliations:** ^1^Unit of Clinical Psychology, Faculty of Health Sciences, King Juan Carlos University, Madrid, Spain; ^2^Laboratory of Neuropsychophysiology, Faculty of Psychology and Sciences, University of Porto, Porto, Portugal

**Keywords:** emotion, pain, unconscious emotion, negative images, attentional capture

There is an error in the Funding statement. The correct name of the Funder is Institute of Health Carlos III of Spain (ISCIII-Co-financed from the European Regional Development Fund: ERDF). The authors apologize for this error and state that this does not change the scientific conclusions of the article in any way.

The original article has been updated.

## Conflict of interest statement

The authors declare that the research was conducted in the absence of any commercial or financial relationships that could be construed as a potential conflict of interest.

